# Periplocin Overcomes Bortezomib Resistance by Suppressing the Growth and Down-Regulation of Cell Adhesion Molecules in Multiple Myeloma

**DOI:** 10.3390/cancers15051526

**Published:** 2023-02-28

**Authors:** Abdul Aziz, Haiqin Wang, Yanpeng Wang, Zhenzhen Li, Chaoying Yang, Zekang Ma, Xiaojuan Xiao, Jing Liu

**Affiliations:** Department of Hematology, The Second Xiangya Hospital, Molecular Biology Research Center, School of Life Sciences, Hunan Province Key Laboratory of Basic and Applied Hematology, Central South University, Changsha 410011, China

**Keywords:** multiple myeloma, periplocin, BTZ-resistance, MM stemness, cell adhesion molecules

## Abstract

**Simple Summary:**

Multiple myeloma (MM) is a hematologic disorder that is incurable and relapses due to its resistance capability towards bortezomib (BTZ). Our study aimed to overcome the BTZ resistance problem of MM by exploring an anti-MM agent. In this study, we reported the significant inhibition of MM growth upon Periplocin (PP) treatment by using MM wild-type (ARP1) and BTZ-resistant type (ARP1-BR) cell lines. We found the significant induction of apoptosis, inhibition of proliferation, suppression of MM stemness, and reduced cell migration ability of MM by PP treatment. Moreover, cell adhesion molecules (CAMs) were investigated and verified as the molecular targets of PP in MM, which were previously reported as crucial for BTZ resistance in MM. Moreover, PP significantly suppressed the growth of tumors in vivo in the MM irrespective of BTZ resistance. PP is a useful natural anti-MM compound to overcome BTZ resistance and downregulate CAMs in MM.

**Abstract:**

Multiple myeloma (MM) is an incurable hematological malignant disorder of bone marrow. Patients with MM receive multiple lines of chemotherapeutic treatments which often develop bortezomib (BTZ) resistance and relapse. Therefore, it is crucial to identify an anti-MM agent to overcome the BTZ resistance of MM. In this study, we screened a library of 2370 compounds against MM wild-type (ARP1) and BTZ-resistant type (ARP1-BR) cell lines and found that periplocin (PP) was the most significant anti-MM natural compound. We further investigated the anti-MM effect of PP by using annexin V assay, clonogenic assays, aldefluor assay, and transwell assay. Furthermore, RNA sequencing (RNA-seq) was performed to predict the molecular effects of PP in MM followed by verification through qRT-PCR and Western blot analysis. Moreover, ARP1 and ARP1-BR xenograft mice models of MM were established to confirm the anti-MM effects of PP invivo. The results showed that PP significantly induced apoptosis, inhibited proliferation, suppressed stemness, and reduced the cell migration of MM. The expression of cell adhesion molecules (CAMs) was suppressed upon PP treatment in vitro and in vivo. Overall, our data recommend PP as an anti-MM natural compound with the potential to overcome BTZ resistance and downregulate CAMs in MM.

## 1. Introduction

Multiple myeloma (MM) is a plasma cell malignancy that is characterized by intraconal genetic heterogeneity and accounts for 10% of all hematological disorders [1]. The proliferation of clonal plasma cells with abnormal origin in the bone marrow microenvironment (BM) leads to a progression of various complications, including the inevitable feature of relapse with no cure and, eventually, death [2]. However, efficient therapeutic regimens have made dramatic progress in treatment options for MM as compared to other cancer types [3,4]. The first approved proteasome inhibitor bortezomib (BTZ) for cancer treatment has been considered the crucial regimen for treating MM patients [5,6]. However, the current chemotherapeutic options including BTZ for the treatment of MM are still facing challenges of relapse due to the refractory feature of MM [7,8,9,10,11]. Therefore, it is necessary to continue the constant developmental progress in treatment options for MM to find novel effective therapeutic agents or strategies to cure MM patients.

The BM is a diverse system of different cell types, soluble factors, adhesion molecules, and signaling pathways [12,13]. The interaction of MM cells with various adhesion molecules and extracellular matrix in BM supports MM pathogenesis, drug resistance, and cell migration [14,15]. In MM pathogenesis, cell adhesion molecules (CAMs) induce cell-adhesion-mediated signaling that mainly leads to the drug resistance features of MM [16]. The participation of CAMs in drug resistance of MM was initially reported by the resistance of MM cells against melphalan and doxorubicin treatments due to the adhesion of fibronectin (FN) to MM cells via cell adhesion molecules CD49d and CD49e [17]. The participation of CAMs in cancer stemness and drug resistance of MM has been reported in the last decades. Various studies have reported that CAMs, including CD44, CD138, ICAM-1 [18,19,20,21,22], VCAM-1 [23], CD11A, CD49D [21,24], α4β1 [25],and CXC motif chemokine receptors (CXCR) integrin [26], are associated with adhesion of MM cells to bone marrow stromal cells (BMSC), which leads to MM stemness and participate in BTZ resistance of MM. However, proper inhibiting or degrading agents against CAMs have still not been discovered to overcome the BTZ resistance capability of MM. Thus, a chemotherapeutic agent which can inhibit the growth of MM to overcome BTZ resistance through molecular targets as CAMs can be a potential regimen to develop the current treatment options in the future.

Chemotherapies that use natural compounds in anticancer regimens often originate the basis for effective development in anticancer treatment options. Exploring natural compounds against cancer is the most common way to novel anticancer drug discovery [27]. Over decades, various natural compounds have been investigated for the effective development of anticancer therapies and many of them have been approved for integration into modern medicine [28,29]. Currently, 25% of the approved anticancer agents originated from natural compounds, 38% were imitated from organic synthesis, and even the existing commercial anticancer drugs are more than 60% from natural origin [27,30]. Therefore, the identification of potential natural anticancer compounds with a different mechanism of action or biological activity might play a role in novel drug discovery against MM progression.

To elevate the treatment progress for the survival rate of MM patients against BTZ resistance, we processed a library of 2370 natural bioactive compounds. After extensive screening, we explored periplocin (PP) as the most effective compound against MM. On further screening of PP against MM, we also found the anti-MM effect of PP in different genetic backgrounds of MM cell lines, in CD138^+^ plasma cells of MM patients, and finally in vivo in MM-affected NCG xenograft mice. PP is a natural compound with anticancer capabilities that is derived and purified from the Cortex Periplocae [31]. Some studies have reported that PP possesses the anticancer competence to inhibit the growth of cancers by affecting EGR1/ERK1/2 and activation of AMPK-mTOR signaling, while downregulatingStat3, AKT/ERK, nuclear factor kappa B, Wnt/catenin signaling pathways and suppressing surviving, c-myc, and Nrf2 expression [32,33,34,35,36,37,38]. However, the anticancer effect of PP has not yet been reported to overcome the BTZ resistance of MM and to investigate its molecular therapeutic targets in MM, which could be a novel regimen for the development of MM treatment options.

In this study, we explored PP for the first time as a strong natural anti-MM agent which significantly induced apoptosis and inhibited the BTZ resistance of MM in vitro and in vivo. The anti-MM effect of PP was associated molecularly with the suppression of CAMs, which were identified from the results of transcriptome sequence analysis. CAMs targeted by PP were then further verified at mRNA and proteins level in MM wild-type ARP1 cell lines and BTZ-resistant type ARP1-BR cell lines and in vivo. This finding provided a crucial potential novel therapeutic agent in the field of myeloma.

## 2. Materials and Methods

### 2.1. Chemicals and Antibodies

Periplocin was purchased from Selleck Chemicals (Houston, TX, USA), and dimethyl sulfoxide (DMSO) was purchased from Sigma-aldrich (St. Louis, MI, USA) to dissolve PP. Cell Counting Kit-8 (CCK-8) was purchased from Vazyme Biotech Co., (Nanjing, China) for cell viability assay. Annexin V-FITC/PI staining kit and Aldefluor reagent were purchased from BD Biosciences (Bergen, NJ, USA) and Stem Cell Technologies (Vancouver, BC, Canada), respectively, for flow cytometric analysis. The antibodies CD2, CDH5, and NRCAM were purchased from Abclonal Technology (Woburn, MA, USA), while GAPDH antibodies were purchased from Santa Cruz Biotechnology (Dallas, TX, USA).

### 2.2. Cells and Cell Culture

MM cell lines from different genetic backgrounds including ARP1 (human IgAκ MM cell lines), ANBL6 (human IL6 dependent MM cell lines, mutation on TP53 gene), RPMI8226 (human MM cell lines with E285K missense mutation on TP53 gene), MM.1S and KMS11 (human MM cell lines with a mutation on TRAF3 gene), ARP1-BR (human IgAκ BTZ-resistant MM cell lines), and ANBL6-BR (human IL6 dependent BTZ-resistant MM cell lines, mutation on TP53 gene) cells were purchased from the American Type Culture Collection (ATCC, Manassas, VA, USA) and provided by the Institute of Hematology and Blood Disease Hospital (IH), Chinese Academy of Medical Sciences, Tianjin, China. The human peripheral B lymphocytes (B cells) were provided by the Laboratory of Medical Genetics, School of life sciences, Central South University, Changsha, China. All the MM cell lines were cultured in RPMI-1640 medium (Gibco, Waltham, MA, USA). The medium used for cell culture of MM cell lines was supplemented with 10% of fetal bovine serum (FBS) (Gibco, Waltham, MA, USA), 100 U/mL penicillin, and 100 μg/mL streptomycin (Thermo Fisher Scientific, Waltham, MA, USA), and kept in condition in 5% CO_2_ at 37 °C. The B cells were cultured in the same medium but supplemented with 15% FBS in the same condition.

### 2.3. Patient Samples and CD138^+^ Cells Isolation

Bone marrow aspirates of MM patients were provided by the Xiangya Hospital of Central South University under the standard procedure of the Ethics Committee of the School of life sciences, Central South University, Changsha, China. Lymphocyte separation fluid was used to isolate the bone marrow mononuclear cells (BMMC) from the provided MM patient’s bone marrow aspirates. A Human CD138 enrichment kit (CD138^+^ Plasma Cells Iso. Kit) was purchased from MiltenyiBiotec (BergischGladbach, Germany) which was used to isolate the primary CD138^+^ cells from BMMC. Peripheral blood mononuclear cells (PBMC) were obtained under standard procedures from healthy donors.

### 2.4. Cell Viability

Cell viability of the related cells were measured through CCK-8 assay by using CCK-8 solution (Vazyme) following the manufacturer’s procedures. An equal number of cell lines were pretreated with PP for 24 h with different concentrations. The PP-treated cell lines were then seeded in a 96-well plate and added CCK-8 solution of 10 μL to each well of seeded cells. The cell lines were incubated at 37 °C and 5% CO_2_ condition for 3 h. The absorbance was measured at 450 nm through a microplate reader.

### 2.5. Colony Forming Assay

An equal number of MM BTZ-sensitive cell lines ARP1 and BTZ-resistant type ARP1-BR cell lines were cultured in a 12-well plate (three thousand cells per well). The related cell lines were cultured in soft agar (Thermo Fisher Scientific, Waltham, MA, USA) and treated for 3 weeks with different concentrations of PP. MyeloCult TM H5100 medium (two drops twice a week) was added by resuspending in the agar of 0.33%. After three weeks, the photos of the 12-well plate were taken and the numbers of colonies were counted through ImageJ (National Institutes of Health, Bethesda, MD, USA) after being scanned under a microscope.

### 2.6. Flow Cytometry Analysis

Apoptosis of ARP1 and ARP1-BR cell lines was measured after 24 h of PP treatment by using an annexin V-FITC/PI staining kit (BD Bioscience, Franklin Lakes, NJ, USA). The cells were processed and stained by following the manufacturer’s procedures and evaluated apoptosis through FACS Calibur (BD Biosciences, Franklin Lakes, NJ, USA) flow cytometer. Similarly, the ALDH proportion with related MM cell lines was measured through a flow cytometer by using an aldefluor reagent, while diethylaminobenzaldehyde (DEAB) was used as a negative control.

### 2.7. Migration Assay

Inhibition of migration ability of the cell lines was identified by chamber migration assay by using transwell chambers (8 μm; BD Biosciences, Franklin Lakes, NJ, USA) in a 24-well plate. Serum-starved ARP1 and ARP1 cells (5 × 10^4^ cells) were pretreated with PP at mentioned concentration for 24 h. The cell lines were washed two times with phosphate-buffered saline (PBS) (Sigma-Aldrich, St. Louis, MI, USA) after PP treatment and were then seeded in upper transwell chamber with 200 μL of FBS-free medium, whereas 600 μL medium supplemented with 15% FBS in the lower was used as a chemoattractant. After incubation at 37 °C and 5% CO_2_ condition for 12 h, the non-migrated cells were discarded through cotton swabs, while the migrated cells were fixed using 3.7% paraformaldehyde (cold) and were then counted manually.

### 2.8. Transcriptome Sequencing

To investigate the molecular effects of PP in MM, RNA sequencing (RNA-seq) of PP-treated ARP1 and ARP1-BR cell lines was performed. The cell lines ARP1 and ARP1-BR were pretreated with PP for 24 h at concentrations of 0 μM and 0.5 μM for RNA-seq. The differentially expressed genes were shown by differential gene volcano map and Venn diagram to determine the estimated molecular effects in RNA-seq of the related cell lines. Kyoto Encyclopedia of Genes and Genomes (KEGG) analysis was used to predict the potential effect of PP in RNA-seq. The DcokThor server was used to dock the PP drug with co-effected molecules predicted by KEGG enrichment analysis in the RNA-seq of both cell lines.

### 2.9. Quantitative Real-Time PCR

Total RNA was extracted from PP pretreated ARP1 and ARP1-BR cell lines by using TRIzole reagent (Vazyme, Nanjing, China) and following the standard procedure. The isolated RNA was purified from DNA by using a gDNA wiper mix (Vazyme, Nanjing, China). The cDNA was produced by reverse transcription of isolated RNA by using HiScript^®^ Q R SuperMix(Vazyme, Nanjing, China). A qRT-PCR kit (HiScript-II One-Step RT-PCR) was purchased from Vazyme (Nanjing, China). A Mastercycler^®^ ep realplex system was used to perform qRT-PCR and determined the final results. The expressions of the target genes at the mRNA level were calculated by the 2^−ΔΔCT^ method. Primers used in qRT-PCR against CDH5, NRCAM, CD2, and GAPDH are listed in Table S1.

### 2.10. Proteins Extraction and Western Blot

Pretreated ARP1 and ARP1-BR cell lines were used to obtain the total cell lysates by utilizing RIPA buffer (Scientific Thermo Fisher, Waltham, MA, USA). All the procedures were done in the presence of protease inhibitors as well as phosphatase inhibitors, according to the manufacturers’ instructions. The proteins were extracted and quantified by A Bicinchoninic Acid Protein Assay Kit purchased from Thermo Fisher Scientific, Waltham, MA, USA to extract proteins and quantified. After quantification, the required amount of protein samples was added to the wells of SDS-PAGE to separate the protein followed by transferring the SDS-PAGE gel to a nitrocellulose membrane (Santa Cruz). The blotted nitrocellulose membranes were incubated with the targeted primary antibody overnight at 4 °C. Next, the nitrocellulose membranes were incubated with a secondary antibody conjugated with HRP for 1 h at room temperature after washing three times with PBS+ 0.05% Tween-20 (Sigma-Aldrich, St. Louis, MI, USA) by incubation with secondary antibody followed by conjugation with HRP. The final results were analyzed by taking the images through GeneGenious Bio-imaging System (Bio-red). Original blots can be found in Supplementary File S1.

### 2.11. Xenograft Mouse Model

To verify the anti-MM effect of PP in vivo, we established MM xenograft mice models by inoculating MM cell lines in vivo. For this purpose, we used NCG xenograft mice (having the characteristic of deficient functional T, B, and NK cells) which were purchased from Gempharmatech Co. Ltd., Jiangsu, China. All the in vivo experiments were performed under the approved standard procedures of the Animal Care and Ethics Committee of Central South University. The mice were subcutaneously inoculated in the flank with 2 × 10^6^ wild-type ARP1 and 2 × 10^6^ BTZ-resistant type ARP1-BR cell lines in 100 μL of PBS. Mice were randomly divided into 4 groups (ARP1-inoculated mice for PP treatments, ARP1-inoculated mice for DMSO as a control, ARP1-BR-inoculated mice for PP treatments, ARP1-BR-inoculated mice for DMSO as a control). PP treatments (5 mg/kg/d) for PP-treatment groups and DMSO for control groups were started after the appearance of the measurable tumors. Tumor volume and body weights of mice were measured daily. A caliper was used to measure the tumor sizes following to calculate the tumor volume by the equation as = length × width 2/2. The mice were euthanized and were imaged at the end of the experiments. Tumors were flaked off from euthanized mice, their weight was measured according to the group category and were then imaged after fixing with 10% formaldehyde. The tumors were processed under standard procedure to further verify the anti-MM target of PP in vivo.

### 2.12. Statistical Analysis

The resulted data were shown in bar graphs by using GraphPad Prism 8 software (GraphPad Software, San Diego, CA, USA). All the values are represented as the mean ± SD. The unpaired two-tailed *T*-test was used to analyze the differences. The values were considered statistically significant at *p* < 0.05, whereas the more significant values were represented as * *p* < 0.05, ** *p* < 0.01, *** *p* < 0.001.

## 3. Results

### 3.1. Periplocin Is an Effective Anti-MM Natural Compound

**Figure 1:**
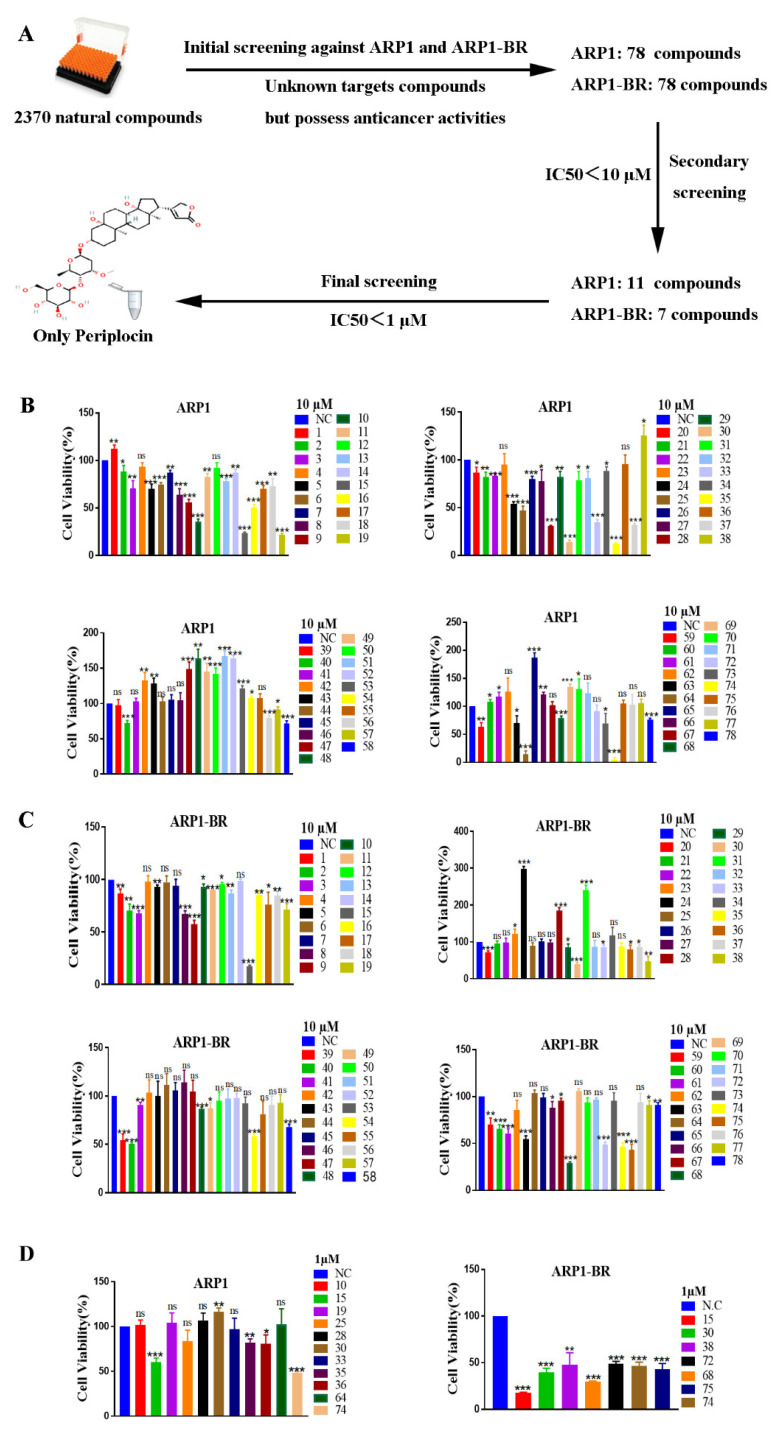
Periplocin is an effective anti-MM natural compound. (**A**) Schematic diagram of PP identification as the most significant anti-MM agent among 2370 anticancer compounds against MM wild type ARP1 and MM BTZ-resistant type ARP1-BR cell lines. (**B**) Effect of 78 drugs on cell viability of ARP1 through CCK-8 assay at a concentration of 10 μM, 11 drugs had IC50 < 10 μM for ARP1 (the number in figures represents a drug; No. 74 represents PP). (**C**) Effect of 78 drugs on cell viability of ARP1-BR through CCK-8 assay at a concentration of 10 μM, 7 drugs had IC50 < 10 μM for ARP1-BR including PP. (**D**) Effect of 7 drugs on cell viability of ARP1 and 11 drugs effect on cell viability of ARP1-BR through CCK-8 assay at a concentration of 1 μM, only PP had had IC50 < 1 μM on both types of cell lines.The bar graphs demonstrated a mean ± SD of three independent experiments. The results were considered significant statistically at *p* < 0.05. The more significant values were demonstrated as * *p* < 0.05, ** *p* < 0.01, *** *p* < 0.001, and ns: not significant.

A library of natural compounds was screened by CCK-8 assay to explore a strong anti-MM natural compound. The library contained 2370 compounds (from Selleck) that possessed anticancer activity [39,40]. After extensive screening, 78 natural anticancer compounds including periplocin were figured out as the most significant anti-MM agents among them against the MM wild-type ARP1 cell lines and MM BTZ-resistant type ARP1-BR at concentrations of 10 μM to 1 μM (Figure 1A). On further screening of 78 natural anticancer compounds form 2370 compounds, 11 compounds were identified as strong anti-MM agents against ARP1, while 7 compounds were identified as strong anti-MM agents against ARP1-BR by having IC50 < 10 μM (Figure 1B,C). Finally, we identified PP (PP, No. 74) as the most effective anti-MM agent among all 2370 compounds with an IC50 < 1 μM against ARP1 and ARP1-BR cell lines (Figure 1D).

### 3.2. Periplocin Inhibits Proliferation and Induces Apoptosis in MM

**Figure 2:**
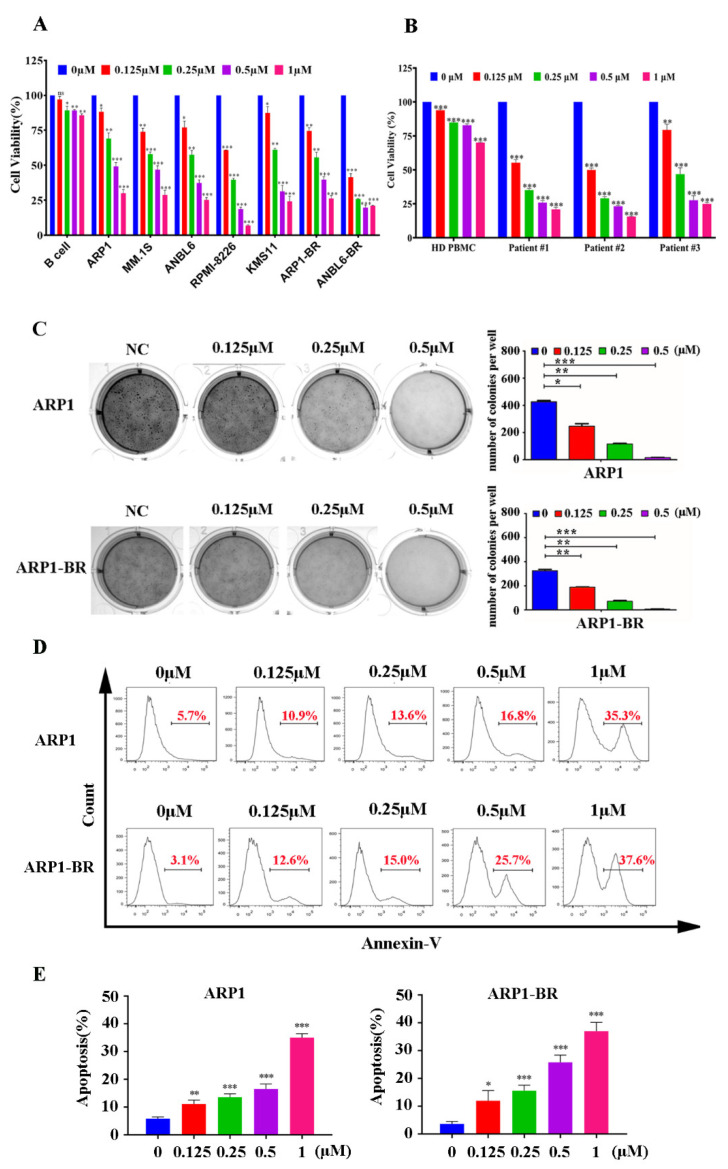
Periplocin inhibits proliferation and induces apoptosis in MM. (**A**) Effect of PP on cell viability of MM cell lines of different genetic backgrounds and B-cell lines with the mentioned concentrations for 24 h through CCK-8 assay. The survival rate of 0 μM PP-treated cells was set to 100%. (**B**) Effect of PP on cell viability of primary CD138^+^ cells isolated from MM patients with indicated concentrations for 24 h through CCK-8 assay. (**C**) Effect of PP on colony-forming abilities of ARP1 and ARP1-BR cell lines treated with the mentioned concentrations of PP for 24 h by using clonogenic assay. The bar graphs represent the number of colonies. (**D**) The changes in apoptosis rate of ARP1 and ARP1-BR cells after treatment with PP with the mentioned concentrations for 24 h, were achieved through flow cytometry using Annexin V stain. (**E**) The bar graph representation of PP-induced apoptosis of ARP1 and ARP1-BR cell lines. The bar graphs demonstrated a mean ± SD of three independent experiments. The results were considered significant statistically at *p* < 0.05. The more significant values were demonstrated as * *p* < 0.05, ** *p* < 0.01, *** *p* < 0.001, and ns: not significant.

To evaluate the effect of PP on the cell viability of MM, seven MM cell lines with different genetic backgrounds (ARP1, ANBL6, MM.1S, RPMI-8226, KMS11, ARP1-BR, and ANBL6-BR), and B cells were treated with various concentrations of PP for 24 h to examine cell viability through CCK-8 assay. The MM cell viability was significantly reduced by PP treatment at mentioned concentrations, while PP had not affected the cell viability of B cells at the same concentration significantly (Figure 2A). Meanwhile PP significantly reduced the cell viability of cells (CD138^+^) isolated from MM patients but did not affect the PBMCs isolated from healthy donors (Figure 2B). These results indicate that PP selectively inhibited the cell viability of MM. Furthermore, to evaluate the long-term effect of PP on MM cell lines proliferation, colony forming assay was performed by using ARP1 and ARP1-BR cells. The results showed that PP suppressed the ability of ARP1 and ARP1-BR cells to form colonies and stopped their proliferation (Figure 2C,D). Next, we used annexin V-FITC and PI staining to examine the effect of PP on apoptosis in MM. Flow cytometry analysis showed that PP induced and increased apoptosis rate in both types of ARP1 and ARP1-BR cells MM cell lines (Figure 2E,F). These results showed that PP can induce apoptosis in MM. Overall these results demonstrated that PP has a strong anti-MM effect by selectively inhibiting MM cell growth and inducing MM cell lines apoptosis irrespective of BTZ sensitivity or BTZ-resistance.

### 3.3. PeriplocinSuppresses Stemness and Cells Migration of MM

**Figure 3:**
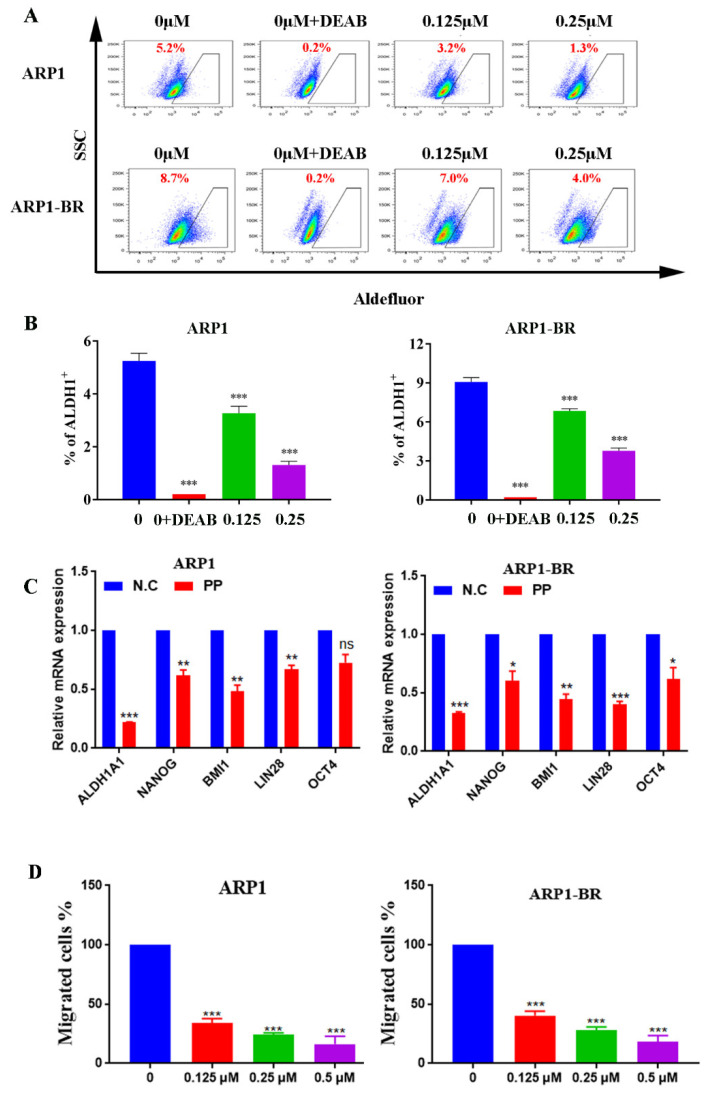
Periplocin suppresses the stemness and cell migration of MM cells. (**A**) Detection of changes in ALDH ratio of ARP1 and ARP1-BR cells by aldefluor assay after PP treatment at mentioned concentrations for 24 h, the gate of the experimental group was determined by using DEAB was used as a control strain. (**B**) Bar graph representation of the decreased ALDH ratio of ARP1 and ARP1-BR cells by PP treatment. (**C**) Detection of downregulation in the expression of ALDH1A1 gene and iPSC genes by qRT-PCR after PP treatment at mentioned concentrations for 24 h. Expression of genes in cells with at 0 μM PP treatment was set to 100%. (**D**) The effect of PP on cell migration ability of ARP1 and ARP1-BR cells through transwell assay at mentioned concentrations for 24 h. The number of migrated cells of 0 μM PP-treated cells was set to 100%.The bar graphs demonstrated a mean ± SD of three independent experiments. The results were considered significant statistically at *p* < 0.05. The more significant values were demonstrated as * *p* < 0.05, ** *p* < 0.01, *** *p* < 0.001, and ns: not significant.

It has been reported that MM stemness is the main feature of recurrence and drug resistance of MM. In this regard, the aldehyde dehydrogenase (ALDH) and induced pluripotent stem cells (iPSC) genes i.e., NANOG, BMI1, LIN28, and OCT4 account for an effective participant in MM stemness [41,42,43]. So, next, we examined the effect of PP on the ALDH ratio in ARP1 and ARP1-BR cells. The changes in ALDH ratio in both cell lines upon PP treatment were measured by using Aldefluor assay through flow cytometry. The results showed that the ALDH ratio was decreased by PP treatment in both types of MM cell lines (Figure 3A,B). In addition, then we verified the changes in the expression level of ALDH1A1 genes and iPSC genes at mRNA level under PP treatment in ARP1 and ARP1-BR cell lines. We found the reduced expression of ALDH1A1 genes and iPSC genes (NANOG, BMI1, LIN28, and OCT4) significantly at the mRNA level in both types of MM cell lines after PP treatment (Figure 3C). These results indicate that PP has the competence of suppressing the stemness of MM. Furthermore, it has been reported that MM cells interact with extracellular matrix and adhesion molecules in BM, which supports MM drug resistance and metastasis of MM. Therefore, assuming the phenomena, we checked whether PP affects the migration ability of ARP1 and ARP1-BR cells. As expectedly, the transwell assay results showed a significant suppression in the migration ability of these both MM cell lines (Figure 3D). Overall, these results demonstrate that PP has a strong effect on MM stemness by decreasing the ALDH ratio and reducing the expression of iPSC genes, and reducing migration ability of MM.

### 3.4. PeriplocinDownregulates Cell Adhesion Molecules

**Figure 4:**
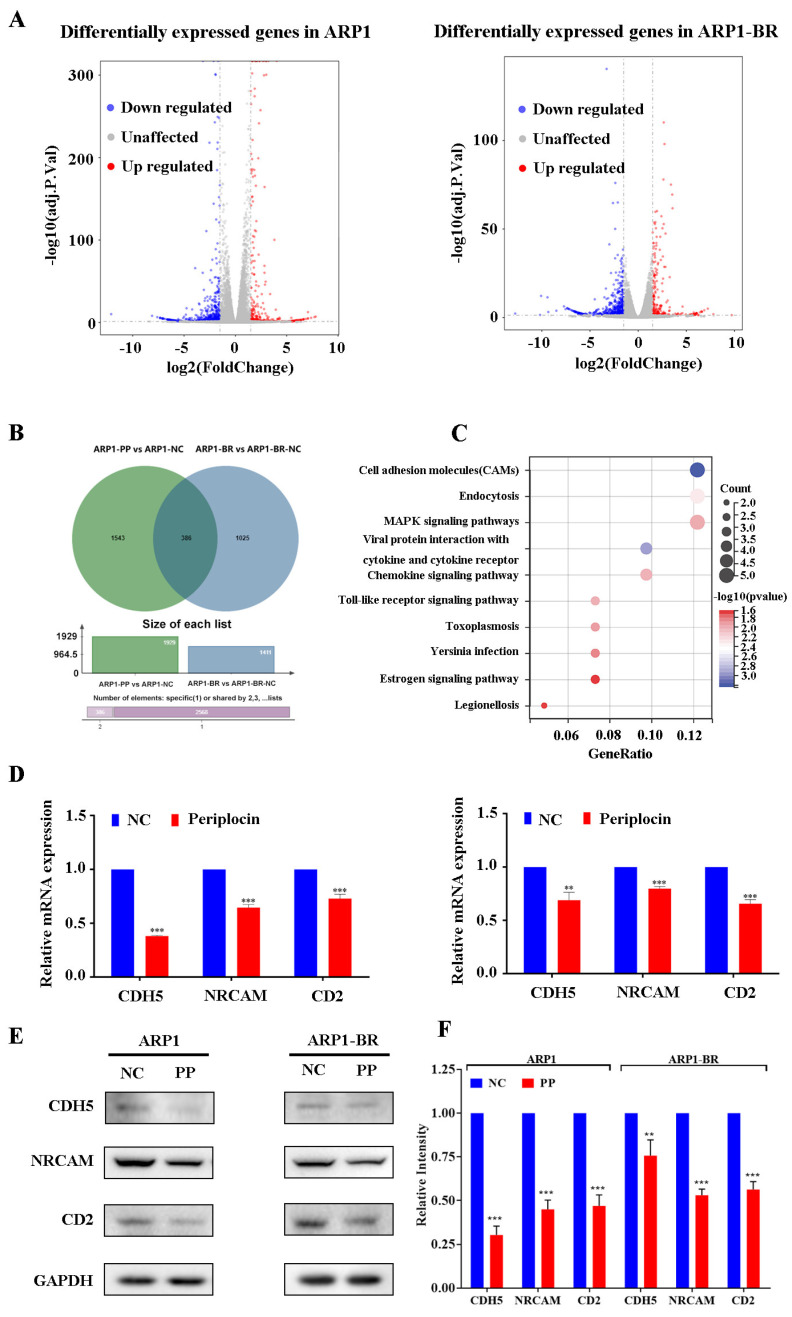
Periplocin downregulates cell adhesion molecules. (**A**) Transcriptome sequence analysis of differentially expressed genes by differential gene volcano map in RNA-sequencing of pretreated ARP1 and ARP1-BR cells with PP at concentrations of 0 μM and 0.5 μM for 24 h. (**B**) Differential gene Venn diagram of transcriptome sequenced of PP pretreated ARP1 and ARP1-BR cells. (**C**) Identification of CAMs as the most significantly co-enriched genes through KEGG pathway analysis in RNA-sequencing of pretreated ARP1 and ARP1-BR cells with PP at a concentration of 0 μM and 0.5 μM for 24 h. (**D**) Identification of significantly reduced expression of CAMs genes CD2, CDH5, and NRCAM at mRNA levels through real-time qRT-PCR in ARP1 and ARP1-BR cell lines at 24 h of PP treatment at a concentration of 0 μM and 0.5 μM. (**E**) The significantly reduced expression of CAMs proteins of CD2, CDH5, and NRCAM genes at protein levels in ARP1 and ARP1-BR cell lines through Western blot in ARP1 and ARP1-BR cell lines at 24 h of PP treatment at a concentration of 0 μM and 0.5 μM.(**F**) Bar graph representation of the significantly reduced expression of CD2, CDH5, and NRCAM at protein levels in ARP1 and ARP1-BR cell lines. The bar graphs demonstrated a mean ± SD of three independent experiments. The results were considered significant statistically at *p* < 0.05. The more significant values were demonstrated as ** *p* < 0.01, *** *p* < 0.001.

To investigate molecular targets of periplocin in MM, transcriptome sequencing was performed for both ARP1 and ARP1-BR cells under 24 h treatment of PP with a concentration of 0 μM and 0.5 μM. The results of the analysis showed that more than 35,000 genes were expressed differentially in both groups of cell lines by using a differential gene volcano map of two-fold changes after PP treatment (Figure 4A). Differential gene Venn diagram analysis of transcriptome sequenced showed 386 genes commonly downregulated in periplocin-pretreated ARP1 and ARP1-BR cells (Figure 4B). Furthermore, KEGG analysis results revealed that Cell Adhesion Molecules (CAMs) were the most co-enriched differential genes in both ARP1 and ARP1-BR cell lines (Figure 4C). As CAMs contribute in cell to cell adhesion and participate in cell migration and metastasis of cancer [44,45], therefore, the prediction of CAMs through KEGG analysis results (Figure 4C) also confirmed the consistency of our initial results (Figure 3D), which showed the suppression in migration ability of ARP1 and ARP1-BR under PP treatments. In addition, the DcokThor server was used to dock the PP drug with CDH5, NRCAM, and CD2, which showed that PP has a high binding affinity with CDH5, NRCAM, and CD2 (Figure S1). Next, looking into transcriptome sequencing analysis results, we verified the predicted changes in CAMs expression level in ARP1 and ARP1-BR cell lines under PP treatment at concentrations of 0 μM and 0.5 μM for 24 h. The qRT-PCR results showed that PP significantly suppressed the expression of CDH5, NRCAM, and CD2 genes at mRNA levels (Figure 4D). Moreover, the significantly reduced expression of CDH5, NRCAM, and CD2 genes at protein levels were also verified through Western blot in ARP1 and ARP1-BR cells after PP treatment at the same concentrations for 24 h (Figure 4E,F).

### 3.5. Periplocin Inhibits Tumor Growth of BTZ-Sensitive Xenograft Mouse Model of MM and Suppresses CAMs In Vivo

**Figure 5:**
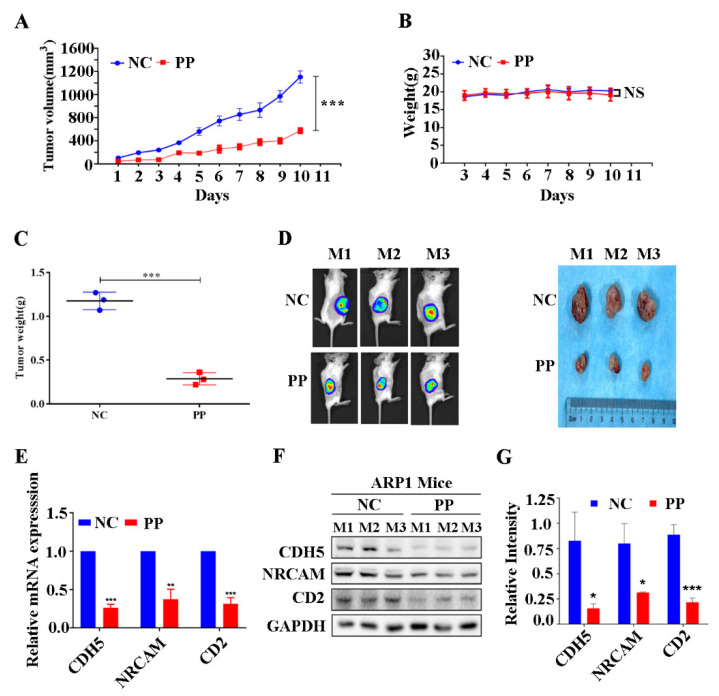
Periplocin attenuates tumor growth in vivo in BTZ-sensitive xenograft mouse model of MM and suppresses CAMs. (**A**) Daily changes in tumor volume of ARP1-inoculated mice after PP treatment, compared with the control untreated group. The data is presented as the mean ±SD from three mice. (**B**) Daily changes in weight of ARP1-bearing mice after PP treatment. (**C**) The changes in weight of tumor created by ARP1-bearing mice after PP treatment. (**D**) The photograph of mice bearing tumor of ARP1 cells after PP treatment before dissection (right side). The photograph of removed tumor from ARP1-cells bearing mice after PP treatment (left). (**E**) Detection in the reduced expression level of CD2, CDH5, and NRCAM genes through qRT-PCR in tissue samples of ARP1-cells bearing mice after PP treatment compared with untreated mice. (**F**) Detection of reduced expression of CD2, CDH5, and NRCAM at the proteins level through Western blot in tissue samples of ARP1-cells bearing mice after PP treatment compared with untreated mice. (**G**) Bar graph representation of the significantly reduced expression of CD2, CDH5, and NRCAM at protein levels in tissue samples of ARP1-cells bearing mice after PP treatment compared with untreated mice.. The results were considered significant statistically at *p* < 0.05. The more significant values were demonstrated as * *p* < 0.05, ** *p* < 0.01, *** *p* < 0.001, and ns: not significant.

Next, we established BTZ-sensitive xenograft (NCG) mouse models of MM by inoculating MM BTZ-sensitive type ARP1 cell lines to evaluate further the effect of periplocin in vivo. After palpation of the measurable tumor under the skin of mice, PP (5 mg/kg/d) was given intraperitoneally. It was observed that PP treatment efficiently suppressed the tumor growth rate of ARP1-inoculated mice as compared to the untreated group (Figure 5A). Fortunately, PP had no adverse effect on the whole body weight of mice models on daily basis (Figure 5B), but had only reduced significantly the weight of created tumor (Figure 5C). Furthermore, the mice whole-body fluorescence imaging showed a relatively low proportion of tumors in PP treated mice group (Figure 5D). In addition, the decreased expression of CD2, CDH5, and NRCAM genes at mRNA and proteins levels by PP treatment were verified through qRT-PCR and Western blot tissue of ARP1-inoculated mice (Figure 5E–G). This data demonstrates the successful attenuation of tumor growth and the decreased expression of cell adhesion molecules CD2, CDH5, and NRCAM by PP treatment in the BTZ-sensitive xenograft mouse model of MM in vivo.

### 3.6. Periplocin Inhibits Tumor Growth of BTZ-Resistant Xenograft Mouse Model of MM and Suppresses CAMs In Vivo

**Figure 6:**
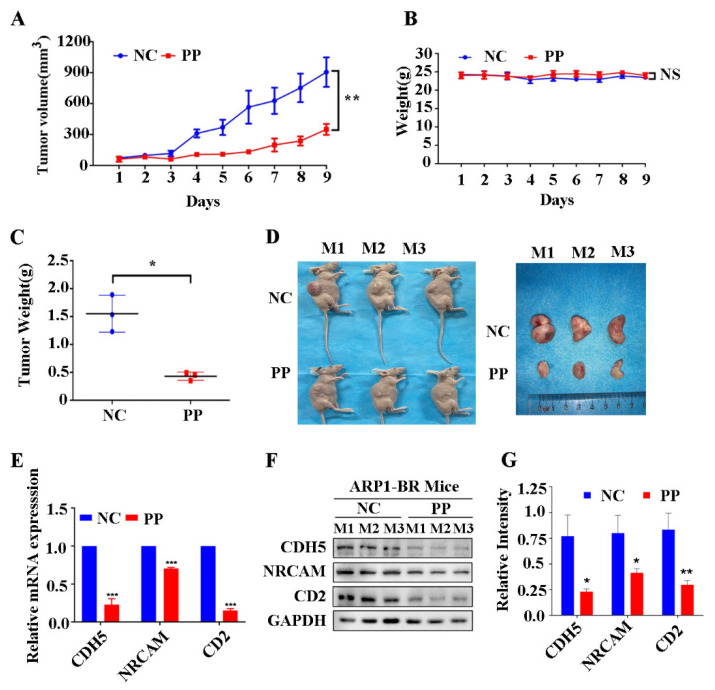
Periplocin attenuates tumor growth in vivo in BTZ-resistant xenograft mouse model of MM and suppresses CAMs. (**A**) Daily changes in tumor volume of ARP1-BR-inoculated mice after PP treatment, compared with the control untreated group. (**B**) Daily changes in weight of ARP1-BR bearing mice after PP treatment. (**C**) The changes in weight of tumor created by ARP1-BR bearing mice after PP treatment. (**D**) The photograph of mice bearing tumor of ARP1-BR cells after PP treatment before dissection (right side). The photograph of removed tumor from ARP1-BR cells bearing mice after PP treatment (left). (**E**) Detection in the reduced expression level of CD2, CDH5, and NRCAM genes through qRT-PCR in tissue samples of ARP1-BR cells bearing mice after PP treatment compared with untreated mice. (**F**) Detection of reduced expression of CD2, CDH5, and NRCAM at the proteins level in tissue samples of ARP1-BR cells bearing mice after PP treatment compared with untreated mice. (**G**) Bar graph representation of the significantly reduced expression of CD2, CDH5, and NRCAM at protein levels in tissue samples of ARP1-BR-cells bearing mice after PP treatment compared with untreated mice. The data is presented as the mean ±SD from three mice. The results were considered significant statistically at *p* < 0.05. The more significant values were demonstrated as * *p* < 0.05, ** *p* < 0.01, *** *p* < 0.001, and ns: not significant.

We also established BTZ-resistant xenograft mouse (NCG) models of MM by inoculating MM BTZ-resistant type ARP1-BR cell lines to evaluate the effect of periplocin in BTZ-resistant NCG xenograft mice in vivo. In this regard, PP (5 mg/kg/d) was given intraperitoneally to mice after the palpation of measurable tumors created by ARP1-BR cells under the skin. As expectedly, PP attenuated significantly the rate of tumor growth and volume of ARP1-BR-inoculated mice (Figure 6A). PP also has no effect on the whole body weight as ARP1-inoculated mice (Figure 6B), but only reduced the size of tumor significantly (Figure 6C). The mice whole-body fluorescence imaging also showed a low proportion of tumor in PP treated mice group (Figure 6D). Furthermore, the decreased expressions of CD2, CDH5, and NRCAM genes by PP treatment were also verified at mRNA and proteins levels through qRT-PCR and Western blot in the tissue of ARP1-BR inoculated mice (Figure 6E,F). This data showed that PP effectively attenuates tumor growth and decreased the expression level of cell adhesion molecules CD2, CDH5, and NRCAM in vivo in BTZ-resistant xenograft mouse model of MM.

## 4. Discussion

Over the past decade, the survival of MM patients has improved with the development of chemotherapy options in the field of myeloma treatment [4]. The well-known effective class of anti-MM drugs such as proteasome inhibitors BTZ has more significant survival and improved response rates [5,6,46]. However, MM patients are still facing major clinical issues of relapse and BTZ resistance due to the complex system of the BM, extracellular matrix, adhesion molecules, and heterogeneous genetic/epigenetic basis of MM [47,48]. The mechanism of recurrence and relapse is poorly understood in MM. However, it has been reported that the BTZ resistance capability of MM leads to recurrence and relapse, making challenges for the effective treatment of MM [7,8,9,10,11,49,50]. Various factors participate in resistance to anti-MM drugs, while many studies have found that CAMs are involved in MM drug resistance [19,20,21,22,23,24,25,26]. Anyhow, BTZ resistance is the most common type of drug resistance in MM and has been identified as a major culprit for the recurrence of MM in most cutting-edge studies. Therefore, the need for novel therapeutic strategies is critical for MM treatment against BTZ resistance to resolve the problem of drug resistance urgently. In this study, we identified PP as the most significant anti-MM agent irrespective of BTZ resistance by screening a library of 2370 anti-cancer natural compounds and exploring its underlying mechanism in MM. We found that PP successfully inhibited proliferation, induced apoptosis, suppressed MM stemness, and reduced the cell migration ability of BTZ-sensitive and BTZ-resistant MM cell lines. Furthermore, we explored the downregulation of CAMs upon PP treatment in both MM cell lines. The growth inhibitory effect of PP was also verified in MM tumors along with the downregulation of CAMs in vivo.

Development in the treatment progress of anti-MM regiments has made impressive achievements and improved outcomes in the field of MM. However, the discovery of novel anti-MM agents, especially from natural sources is considered a crucial approach to MM treatment. Studies have reported that PP is a natural anti-cancer agent which showed the inhibition of tumor cell proliferation and induction of apoptosis in some cancers through different mechanisms on a cellular level in vitro and in vivo [37,51,52,53,54]. Clinically, PP has been used for its cardiotonic effects [55]. A study reported clinical observation of 147 cases of chronic congestive heart failure under the treatment of Xiangjiapi mixture (including ingredients mainly PP), which safely showed high efficacy rates [56]. It has been reported that high doses or prolonged periods of PP usage can cause complications of arrhythmia which could limit its clinical application [55,57]. Anyhow, the combination therapy of PP with TRAIL or PP with panax notoginseng saponins can reduce the cardiotoxicity of PP [55,58]. The contribution of PP in the induction of apoptosis has been reported, but the effect of PP against MM is not reported yet to overcome the relapse and BTZ resistance of MM. Therefore, in the current study, we explored PP as the most significant anti-MM agent in over two thousand natural compounds by suppressing the growth of MM BTZ-sensitive type ARP1 and BTZ-resistant type ARP1 cell lines. Furthermore, we examined the effect of PP through CCK-8 assay on the cell viability of more MM cell lines, B cells, CD138^+^ cells isolated from MM patients, and PBMC from healthy donors. As a result, we found that PP suppressed significantly the cell viability of MM but had no significant effect on B cells or PBMC. These results indicate that PP can selectively inhibit the cell viability of MM. In addition, we further observed the inhibition of proliferation by suppressing the colony-forming ability of ARP1 and ARP1-BR cells in clonogenic assays and also found the PP-induced apoptosis through flow cytometry in related MM cell lines. Next, we studied the characteristics of MM stemness about BTZ-resistance of MM in MM stemness articles and found that ALDH and iPSC genes i.e., NANOG, BMI1, LIN28, and OCT4 are effective participants in MM stemness, which leads to recurrence and drug resistance of MM [41,42,43,59,60,61,62]. Therefore, we performed an Aldefluor assay of ARP1 and ARP1-BR cells to check whether PP affects ALDH or not. As expected, PP decreased the proportion of ALDH ratio in both types of cell lines. In addition, we also found decreased expressions of ALDH1A1 and iPSC genes expression at mRNA level in ARP1 and ARP1-BR cells by qRT-PCR after PP treatment. Overall, these results indicate the inhibition of MM growth and stemness by PP treatment to overcome BTZ resistance of MM cell lines.

Next, we performed the RNA-seq of ARP1 and ARP1-BR cells, which were pretreated with PP at concentrations of 0 μM and 0.5 μM for 24 h to identify the molecular effect of PP in MM. The results of analyses showed by differential gene volcano map and Venn diagram analysis of transcriptome sequenced that more than 35,000 altered genes were found which were differentially expressed after PP treatment, whereas 386 genes were commonly downregulated in RNA-seq analysis of PP pretreated ARP1 and ARP1-BR cells. Especially, the co-downregulation of CAMs was predicted through KEGG enrichment analyses in both ARP1 and ARP1-BR cells. In articles, it has been reported that CAMs are mainly involved in cell adhesion to induce the migration of cancer cells and cancer metastasis [44,45,63,64]. Therefore, the predictions of CAMs like molecules that involve in the migration of cancer cells as downregulated by PP were also speculated initially based on our transwell assay results. As expectedly, the co-downregulation of CAMs results by KEGG enrichment analysis (Figure 4C) were consistent with transwell assay results (Figure 3D), which already showed the inhibition of migration ability of MM cell lines ARP1 and ARP1-BR upon PP treatment.

In previous studies, various researchers have reported that CAMs are involved in cell-adhesion mediated drug resistance of MM [19,20,21,22,23,24,25,26]. It was reported initially based on the adhesion of MM cells to FN through CD49d and CD49e which mediates MM cell survival and anti-apoptosis [17]. Recently, studies also reported the emerging role and close relation of CAMs with cancer drug resistance [19,65,66]. Therefore, keeping in mind the initial results and the prediction of KEGG analysis of ARP1 and ARP1-BR cell lines in the current study, we then examined the effect of PP on the expression level of CAMs including CD2, CDH5, and NRCAM. We found significantly decreased mRNA expression levels of CD2, CDH5, and NRCAM in ARP1 and ARP1-BR cell lines through qRT-PCR after PP treatments. Furthermore, the significantly reduced expression at protein levels of CD2, CDH5, and NRCAM were also verified through Western blot in ARP1 and ARP1-BR cells after PP treatments. In addition, the significantly reduced protein levels of CD2, CDH5, and NRCAM by PP treatment were also verified in the tissue of ARP1 and ARP1-BR cells inoculated xenograft mice. Although PP has been investigated as a strong anti-MM natural compound to overcome BTZ-resistance of MM and downregulate CAMs in both wild-type ARP1 and BTZ-resistant type ARP1-BR in this study, it must be noted that MM BTZ resistance executed by CAMs under PP treatment still needs further investigation for its detailed mechanism.

## 5. Conclusions

Our results suggest that PP is a potential natural anti-MM agent for the significant inhibition of MM growth to overcome the BTZ resistance of MM. Furthermore, CAMs are the molecular target of PP in MM, which were previously reported for cell-adhesion mediated drug resistance. Further investigation is required in the future to find the in-depth mechanism of how PP overcomes BTZ resistance through CAMs to develop this novel regiment in the field of MM. However, in this study, for the first time, we reported that PP significantly attenuate MM growth in both BTZ-sensitive and BTZ-resistant MM cell lines and reduced the expression of CAMs in vitro and in vivo.

## Data Availability

Data are contained within the article and Supplementary Materials.

## Supplementary Materials

The following supporting information can be downloaded at: https://www.mdpi.com/article/10.3390/cancers15051526/s1 Figure S1: The DcokThor server was used to dock Periplocin (PP) drug with CD2, CDH5, and NRCAM; Table S1: qRT-PCR primers. File S1: Original blots.
