# Supplementary material for: Periplocin Overcomes Bortezomib Resistance by Suppressing the Growth and Down-Regulation of Cell Adhesion Molecules in Multiple Myeloma
Source: Cancers (Basel). 2023 Feb 28;15(5):1526. doi: 10.3390/cancers15051526 (PMC10001131; doi:10.3390/cancers15051526)
Supplement: Supplementary file 1 [file cancers-15-01526-s001.zip › File S1.pdf]

Periplocin overcomes bortezomib resistance by suppressing the growth and down-regulation of cell adhesion molecules in multiple myeloma

Western blots details

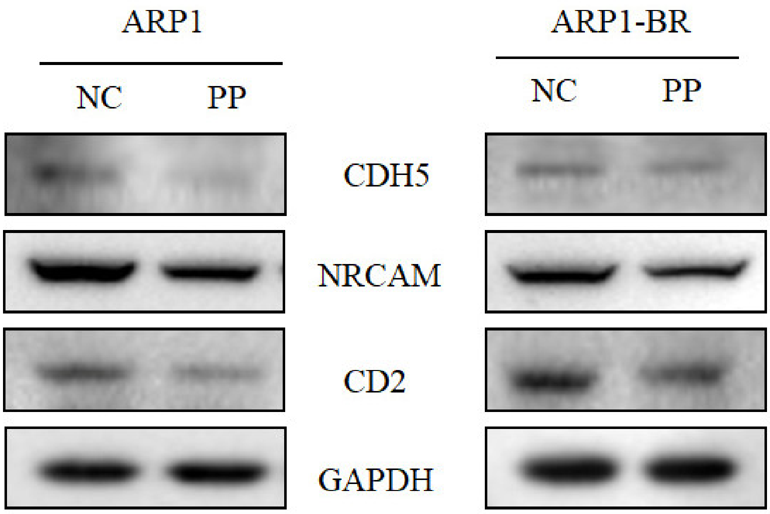

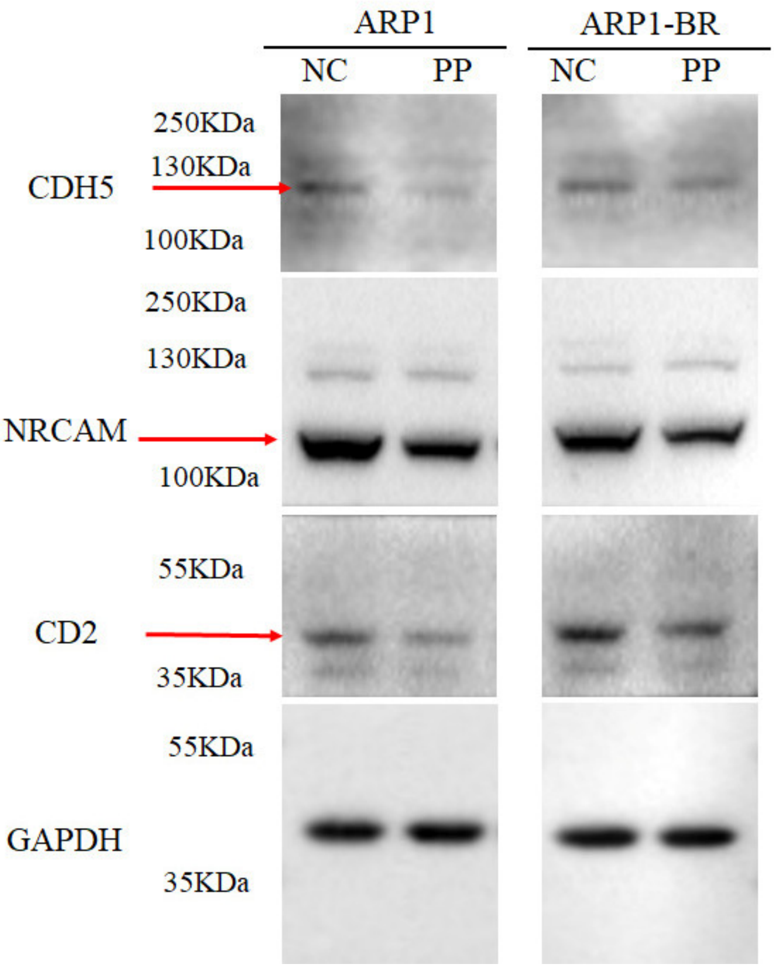

ARP1

CDH5	100:35
NRCAM	100:48
CD2	100:50
GAPDH	100:112

ARP1-BR

CDH5	100:77
NRCAM	100:53
CD2	100:57
GAPDH	100:102

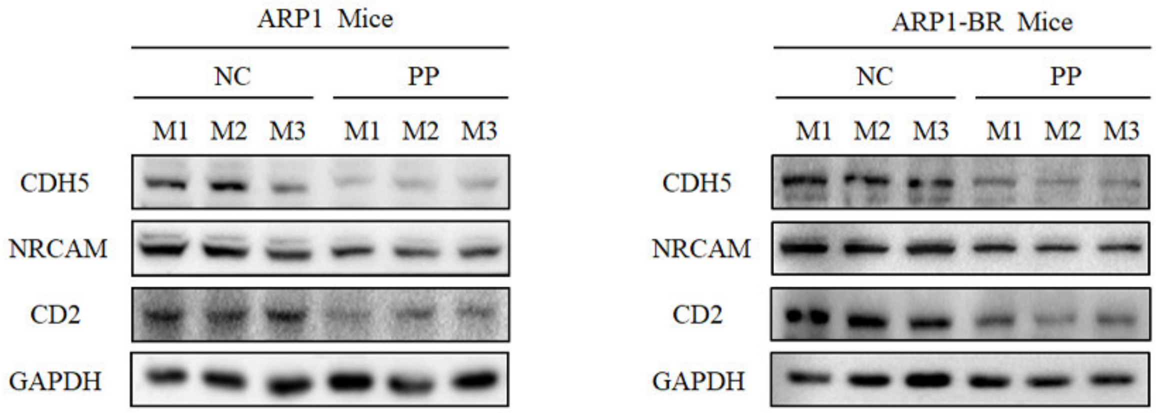

ARP1

CDH5	100:121:68:16:25:24
NRCAM	100:98:83:46:39:46
CD2	100:106:110:25:32:33
GAPDH	100:124:135:148:127:146

ARP1-BR

CDH5	100:91:90:37:32:35
NRCAM	100:91:103:47:42:45
CD2	100:105:102:36:27:33
GAPDH	100:128:149:124:104:98

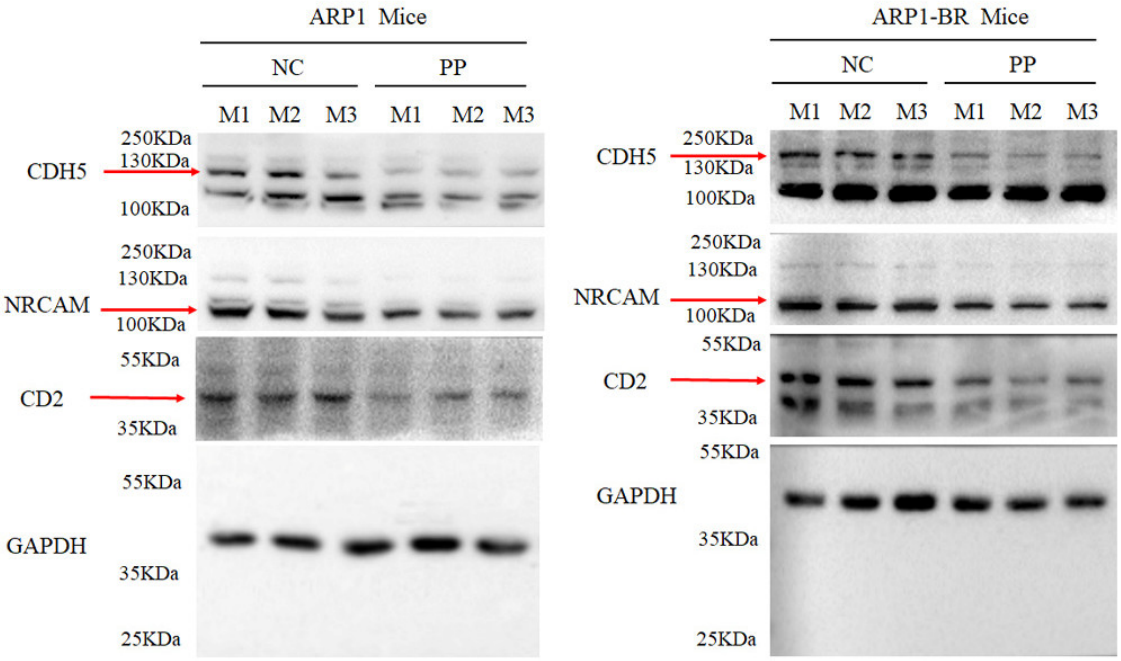

ARP1 Mice
NC
PP
M1 M2 M3 M1 M2 M3
250KDa
130KDa
CDH5
100KDa
250KDa
130KDa
NRCAM
100KDa
55KDa
CD2
35KDa
55KDa
GAPDH
35KDa
25KDa
ARP1-BR Mice
NC
PP
M1 M2 M3 M1 M2 M3
250KDa
CDH5
130KDa
100KDa
250KDa
130KDa
NRCAM
100KDa
55KDa
CD2
35KDa
55KDa
GAPDH
35KDa
25KDa
