# Supplementary material for: Periplocin Overcomes Bortezomib Resistance by Suppressing the Growth and Down-Regulation of Cell Adhesion Molecules in Multiple Myeloma
Source: Cancers (Basel). 2023 Feb 28;15(5):1526. doi: 10.3390/cancers15051526 (PMC10001131; doi:10.3390/cancers15051526)
Supplement: Supplementary file 1 [file cancers-15-01526-s001.zip › SUPPLEMENTARY MATERIALS.pdf]

SUPPLEMENTARY MATERIALS for

Periplocin overcomes bortezomib resistance by suppressing the growth and down-regulation of cell adhesion molecules in multiple myeloma

Supplementary Figure

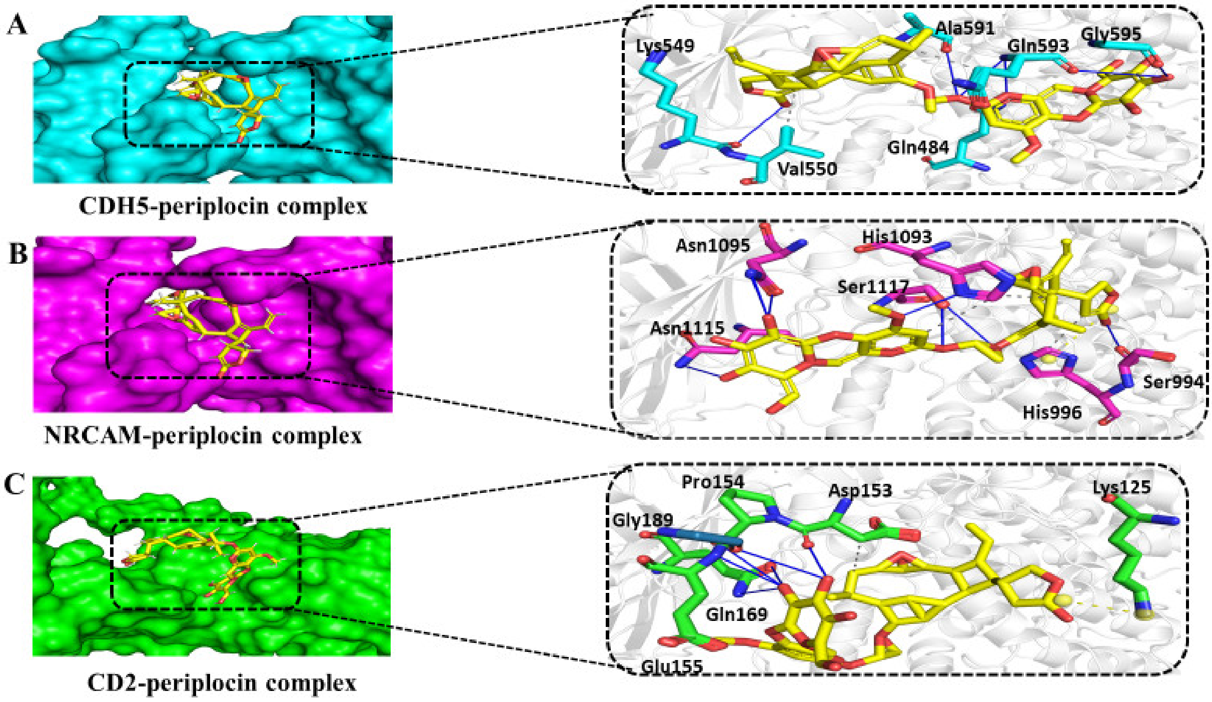

Figure S1

The DcokThor server was used to dock Periplocin (PP) drug with CD2, CDH5, and NRCAM. **(A)** The docking score predicted for the perplocin-CDH5 complex was -2.269 with 3 hydrophobic and 7 hydrogen bonds. The residues involved in hydrogen bonds were GLN484, LYS549, ALA591, GLN593, and GLY595. **(B)** The docking score predicted for perplicin-NRCAM complex was -7.592 with 10 hydrogen bonds, 3 hydrophobic interactions, and 1 salt bridge. The hydrogen bonds formed by the PP-NRCAM complex include SER994, HIS1093, ASN1095, ASN1115, and SER1117 residues while the residues involved in hydrophobic interaction were HIS996 and the salt bridge was HIS996 and HIS1093. **(C)** The DockThor predicted docking score for the PP-CD2 complex was -5.981 and have 7 hydrogens, 1 salt bridge, and 1 hydrophobic bond. The hydrogen

bonds between PP and CD2 were formed by residue ASP153, PRO154, GLU155, GLN169, and GLY189.

Table S1: qRT-PCR primers.

GAPDH-F: 5'-TCGGAGTCAACGGATTTGGT-3'
GAPDH-F: 5'-TGGAATTTGCCATGGGTGGA-3'
CDH5-F: 5'-TCACCTTCTGCGAGGATATGG-3'
CDH5-R: 5'-GAGTTGAGCACCGACACATC-3'
NRCAM-F: 5'-CGAGGCGTCTGAGCAGTATTT-3'
NRCAM-R: 5'-CATTCAAGGGCTTCCACGT-3'
CD2-F: 5'-GGAGTCGGAGAAATGATGAG-3'
CD2-R: 5'-TGGTGGAGGAGGATGTTG-3'
ALDH1A1-F: 5'-TCCTGGTTATGGGCCTACAG-3'
ALDH1A1-R: 5'-CTGGCCCTGGTGGTAGAATA-3'
NANOG-F: 5'-TGAAATCTAAGAGGTGGCAGAA-3'
NANOG-R: 5'-CCTGGTGGTAGGAAGAGTAAAG-3'
BMI1-F: 5'-TTGTTGCAGTGAAGAAAAACCT-3'
BMI1-R: 5'-TTCAGACATAGCAGAAGGCA-3'
LIN28-F: 5'-GAGCATGCAGAAGCGCAGATCAAA-3'
LIN28-R: 5'-TATGGCTGATGCTCTGGCAGAAGT-3'
OCT-4-F: 5'-GGAGGAAGCTGACAACAATGA-3'
OCT-4-R: 5'-CTCTCACTCGGTTCTCGATACT-3'
